# Supplementary material for: Screening of potential regulatory genes in carotid atherosclerosis vascular immune microenvironment
Source: PLoS One. 2024 Dec 9;19(12):e0307904. doi: 10.1371/journal.pone.0307904 (PMC11627393; doi:10.1371/journal.pone.0307904)
Supplement: Attachment — Submitted filename: PONE-D-24-01429_Review-comments_Mamun_20240217.docx [file pone.0307904.s001.docx]

**Title:** *Screening of potential regulatory genes in carotid atherosclerosis*

*vascular immune microenvironment*

**Overall comments:**

Zhang Y et al., studied potential regulatory genes in carotid atherosclerosis vascular immune microenvironment. Identifying potential genes that regulate the progression of carotid atherosclerosis is a demanding research topic. There is relevant published work so far (Zheng K et al., 2023, Dong R et al., 2022, Wang L et al., 2021). This study further extends the contemporary knowledge in carotid atherosclerosis immunology.

Previous studies used same database and identified same hub genes in some extent. For example, Liu C., 2021 identified RBM47, HCK, CD53, TYROBP, and HAVCR2 as Hub Genes using the same Gene Expression Omnibus database (GSE43292). In addition, Yao Yuan et al., 2024 analyzed GSE43292 database and identified hub genes such as AKTIP, ASPN, FAM26E, RAB23, PLS3, and PLSCR4. Ni Jiajun et al., 2023 and Chen M., et al., 2021 were used GSE28829 database and explored Go and KEGG pathways. Therefore, I am unsure how this study is novel compared to what is published so far.

Concern:

I think, there are limitation of ssGSEA and ESTIMATE algorithym

The authors have converted RNA sequencing data to immune cell subtypes using ssGSEA algorithm. However, ssGSEA algorithm have a number of limitations. For example,

Dependency on Predefined Gene Sets, Sensitivity to Noise, Ranking Metric Sensitivity.

The results of ssGSEA can be influenced by the choice of ranking metric used to order genes. Different metrics may lead to variations in enrichment scores and potentially affect the interpretation of results. **Therefore, Interpretability Challenges will be remaining** especially when multiple gene sets show enrichment.

While ESTIMATE scores can be correlated with clinical outcomes, they do not reflect causation.

Minor Correction:

1) I see some of the cases, abbreviations are clearly explained such as DEGs, GO, KEGG. However, still some are left. Those are suggested to be elaborated in the first instance. For example, ssGSEA, ESTIMATE
2) Is it possible to sort the order of the immune cell subtypes fraction based on the p values in Figure 1A and 2A? This will reflect the order of significant fractions?
3) Can you flip the position of CAS and control violin figures to control and CAS. Usually, control comes first. In addition, can you switch the color between control and CAS in figure 1C to match with figure 1B?
