# Supplementary material for: Screening of potential regulatory genes in carotid atherosclerosis vascular immune microenvironment
Source: PLoS One. 2024 Dec 9;19(12):e0307904. doi: 10.1371/journal.pone.0307904 (PMC11627393; doi:10.1371/journal.pone.0307904)
Supplement: Attachment — Submitted filename: Response to Reviewers.docx [file pone.0307904.s002.docx]

Reviewer #1: Zhang Y et al., studied potential regulatory genes in carotid atherosclerosis vascular immune microenvironment. Identifying potential genes that regulate the progression of carotid atherosclerosis is a demanding research topic. There is relevant published work so far (Zheng K et al., 2023, Dong R et al., 2022, Wang L et al., 2021). This study further extends the contemporary knowledge in carotid atherosclerosis immunology.
Previous studies used same database and identified same hub genes in some extent. For example, Liu C., 2021 identified RBM47, HCK, CD53, TYROBP, and HAVCR2 as Hub Genes using the same Gene Expression Omnibus database (GSE43292). In addition, Yao Yuan et al., 2024 analyzed GSE43292 database and identified hub genes such as AKTIP, ASPN, FAM26E, RAB23, PLS3, and PLSCR4. Ni Jiajun et al., 2023 and Chen M., et al., 2021 were used GSE28829 database and explored Go and KEGG pathways. Therefore, I am unsure how this study is novel compared to what is published so far.

Re: Thank you very much for your comments, we understand your concern. First, we added the expression of some hub genes in the carotid atherosclerosis and their correlation with immune cells.These hub genes are completely new, and no previous studies have focused on their role in the carotid atherosclerosis. Secondly, we started to screen the key genes from the immune microenvironment.We have strengthened the correlation between hub genes and the immune microenvironment and disease progression.It's one of our highlights.Thank you very much again for your comments, we benefit from your comments.

Reviewer #2: In this research paper, the authors aim to uncover the changes in the immune microenvironment of vascular tissues at various stages during the progression of carotid atherosclerosis. Additionally, they seek to identify potential hub genes that regulate the immune microenvironment in this pathology.

It is an intersting work ; however, I have some comments and minor revisions :
1. This paper is founded on the data from two research papers (related to GSE43292 and GSE28829), which should be cited as references.

Re: Thank you very much for your comments, we have cited the above two research papers.

2. There is a mistyping in the results section. It should be corrected to "immune score" instead of "immune sore".

Re: Thank you very much for your comments, we apologize for such an error, we have changed.

3. In the results section, the authors reported that "ACTN2 was positively correlated with most immune cell subtypes infiltration" and "PTPRC was negatively correlated with most immune cell subtypes infiltration." However, the discussion section contradicts these findings, stating that "ACTN2 is negatively correlated with most immune cell subtypes infiltration" and "PTPRC is positively correlated with most immune cell subtypes infiltration." To ensure consistency and coherence, the sentence should be corrected to align with the results and discussion.

Re: Thanks for your comments, we have made changes. ACTN2 was negatively correlated with most immune cell subtypes infiltration and PTPRC was positively correlated with most immune cell subtypes infiltration.

4. In Figure 1C, “CAP” should be replaced by “CAS”.

Re: Thank you very much for your comments. We have revised and replaced Figure1 in the manuscript.

5. The legend of Figure 1B and 2B should be corrected as follows: "The differences in immune score between the carotid atherosclerosis and control samples."

Re: Thank you very much for your comments, we have made changes.

6. In the legend of Figure 5A and 5C, abbreviations such as BP (Biological Process) and CC (Cellular Component) should be added.

Re: Thank you very much for your comments, we have added the full names of BP (Biological Process), CC(Cellular Component) and MF (molecular function).

7. In the legend of Figures 5E and 5F, the significance of colors should be specified.

Re: Thank you very much for your comments, we have revised it. Red to yellow indicated that genes rank from high to low in the interaction network.

8. Genes such as ACTN2 and PTPRC should be written in italics.

Re: Thank you very much for your comments, we have revised it.
