# Supplementary material for: Screening of potential regulatory genes in carotid atherosclerosis vascular immune microenvironment
Source: PLoS One. 2024 Dec 9;19(12):e0307904. doi: 10.1371/journal.pone.0307904 (PMC11627393; doi:10.1371/journal.pone.0307904)
Supplement: Attachment — Submitted filename: Response to Reviewers.docx [file pone.0307904.s003.docx]

Reviewer #1: In the revised version, Zhang and colleagues have included three additional figures (Figure 9, 10 and 11). I am wondering, the authors have validated diagnostic efficiency on those two GSE cohorts. Models developed to identify diagnostic genes may be overly complex and tailored to the specific dataset, leading to poor performance when applied to new, unseen data.

Regularization techniques and cross-validation are necessary to mitigate this type of risk. Despite this limitation, the authors have reported novel hub gene that may have a significant impact in the field.

Reply: Thank you very much for your review of our manuscript and your valuable comments. We have learned much from your comments, revised the manuscript, and improved the quality of the manuscript. We agree with you. We verified the diagnostic efficacy in both the GSE43292 cohort (Figure 9) and the GSE28829 cohort (Figure 10). However, as you are concerned, the screening models for diagnostic genes are diverse and complex, and the diagnostic efficacy of the genes we screened is unknown in other datasets. This still requires high-quality cohorts with sufficient sample sizes to validate our results. We did not use regularization techniques and cross-validation. A variety of machine learning algorithms, including regularization techniques and cross-validation, screen key genes and intersect them, which can improve the accuracy and reliability of results. Considering that the number of genes screened by the maximum neighborhood component algorithm is limited, and some genes have been confirmed by previous studies. Therefore, instead of further narrowing the scope by regularization techniques and cross-validation, we directly validated the diagnostic efficacy of all unpublished genes in the data sets. The above shortcomings were supplemented in our discussion. Thank you again for your comments, we have learned a lot from them.

Reviewer #2: Thank you for addressing all my concerns, however, the manuscript still needs to

be revised:

1.First, there are several spelling and grammar errors, so the English must be proofread by a native English speaker.

Reply: Thank you for your comments, we have asked a native English speakers to polish our manuscript.

2.There is a mistyping in the manuscript. It should be corrected to "GSE 28829" instead of "GSE288292".

Reply: Thank you for your comments. We apologize for the error. We have made changes in the manuscript.

3.As suggested by the reviewer, previous studies used the same database and identified some hub genes, such as RBM47, HCK, CD53, AKTIP, ASPN, PLS3, and PLSCR4. In this regard, the results of these papers should be discussed in the manuscript to compare them with the present study and to suggest hypotheses that could explain the differences in hub genes found in these different studies.

Reply: Thank you very much for your comments. We have benefited from your comments. We elaborated on this in our discussion. Previous studies used the same data sets as ours. These studies, including ours, have obtained hub genes that, while partially overlapping, are not completely consistent. This may be related to the inconsistency of the algorithms used. Liu et al. (33519905) identified RBM47, HCK, CD53, TYROBP, and HAVCR2 as Hub Genes in Advanced Atherosclerotic Plaques via Network-Based Analysis. They first used WGCNA to screen key modules, then constructed protein interaction networks and screened out hub genes, all of which were pathogenic genes. Our study first distinguished the immune phenotypes of the samples using consensus clustering. On this basis, differentially expressed genes were screened, and hub genes were screened by maximum neighborhood component algorithm. These hub genes may play an important role in the immune phenotype differentiation of samples. In our study, hub genes were screened from two perspectives of high and low immune cell infiltration, including potential pathogenic and protective genes. Thank you again for your comments, we have benefited from them and have improved the quality of our manuscripts accordingly.
